# Two cases of *Helicobacter pylori* negative mixed early gastric tumors: case report

**DOI:** 10.3389/fmed.2026.1755704

**Published:** 2026-06-26

**Authors:** Xingting Luo, Xiaoyuan Yi, Haixiao Fu, Liang Ye, Bin Huang, En Qin, Bin Li, Xuhua Xiao, Liyan Wang

**Affiliations:** 1 Digestive Department, The Affiliated Hospital of Guilin Medical University, Guilin, China; 2 Department of Gastroenterology, Affiliated Liuzhou People Hospital of Guangxi Medical University, Liuzhou, China

**Keywords:** case report, early gastric cancer, gastric mucous neck cell, gastric phenotype, *Helicobacter pylori* negative, mixed type

## Abstract

Early gastric cancer, which is negative for *Helicobacter pylori* (*H. pylori*), is characterized by difficulties in endoscopic detection, pathological diagnosis, and differentiation. With the deepening understanding of gastric tumors, clinically, there has been a gradual discovery of some early gastric cancers that do not fit conventional categories. This article reports two cases of atypical early differentiated gastric-type adenocarcinoma with mixed features of foveolar epithelial type, mucous neck cell type, chief cell type, and parietal cell type, ultimately diagnosed as a special type of early gastric cancer. We detail the endoscopic features, histopathological findings, and immunohistochemical profiles of these cases, highlighting the diagnostic challenges and the importance of recognizing such rare variants. The identification of these mixed-cell-type adenocarcinomas has significant implications, including expanding the current pathological classification of early gastric cancer, modifying existing endoscopic surveillance strategies, and influencing treatment decisions, such as the extent of endoscopic resection or lymph node dissection. Moreover, these findings underscore the need for heightened awareness among pathologists and endoscopists when encountering *H. pylori*-negative early gastric cancers with ambiguous morphology. By shedding light on this underrecognized entity, our report contributes to more accurate diagnosis and personalized management of early gastric cancer patients.

## Introduction

*Helicobacter pylori* is the most important independent risk factor for the development of gastric cancer through the Correa cascade of gastric malignancy. Approximately 90% of gastric cancers are associated with *H. pylori* infection (1). With improvements in sanitary conditions and increased emphasis on *H. pylori* eradication therapy, the rate of *H. pylori* infection has significantly declined (2). It can be anticipated that the incidence of *H. pylori*-negative gastric cancer will increase relatively. Nevertheless, *H. pylori*-negative gastric cancer remains relatively rare compared to *H. pylori*-positive gastric cancer. Therefore, there is limited research on *H. pylori*-negative gastric cancer. This article presents the clinical and pathological features of two cases of early-stage *H. pylori*-negative gastric cancer, with the aim of providing more clinical experience for the diagnosis of *H. pylori*-negative early gastric cancer.

*Helicobacter pylori* infection causes inflammatory changes in the gastric mucosa. If *H. pylori* is not eradicated on time, it can eventually lead to gastric mucosal atrophy and intestinal metaplasia, which may progress to gastric cancer. Gastric cancer poses a severe threat to human health (3–5). Most gastric cancers are caused by *Helicobacter pylori* infection. However, the incidence of *H. pylori* infection-negative gastric cancer (HpNGC) ranges between 0.42 and 5.40%. Several types of HpNGC have been identified, including intramucosal signet-ring cell carcinoma, gastric fundic gland-type cancer, foveolar-type gastric cancer, cardia gland-type gastric cancer, pyloric gland-type gastric cancer, and mixed-type gastric carcinoma (6, 7).

## Case 1

**Figure 1:**
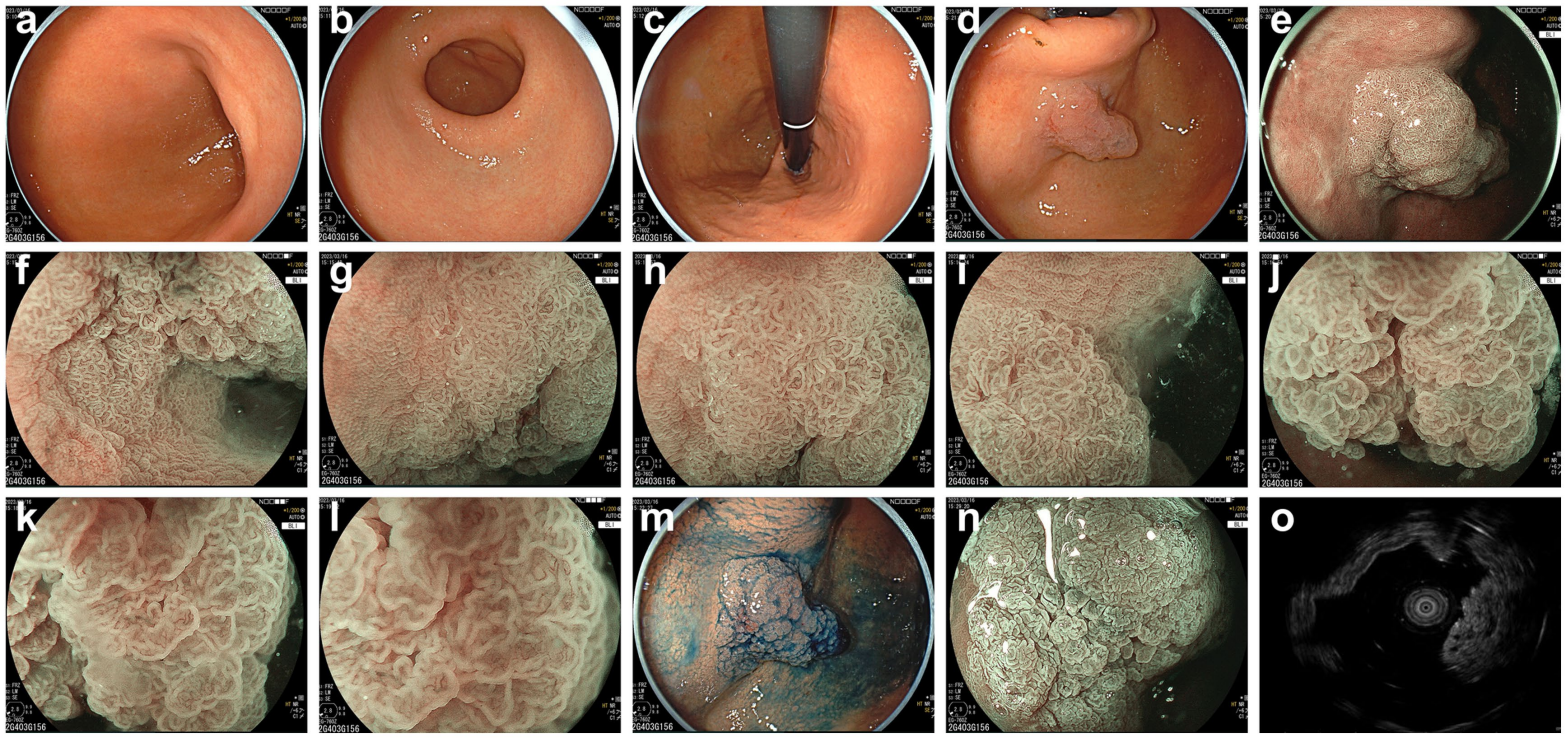
**(a)** White light reveals that the mucosa of the gastric body was smooth, without mucus adhering and diffuse erythema. **(b)** The mucosa of the antrum of the stomach showed no atrophy. **(c)** White light reveals RAC in the background mucosa. **(d)** White light shows a lesion located in the fundic fornix, classified as a 0-IIa + Is-type lesion. **(e)** BLI shows an apricot-colored lesion. **(f–l)** Magnifying endoscopy demonstrates clearly defined lesion borders from the background mucosa, the RAC pattern in the surrounding normal mucosa, and granular and villous changes on the lesion surface. **(m)** Indigo violet staining reveals clear lesion borders and well-defined lesions. **(n)** Acetic acid staining of the lesion. **(o)** Endoscopic ultrasonography findings.

A 53-year-old woman presented with intermittent upper abdominal bloating and pain for 1 year. Both the C13 urea breath test and serum *Helicobacter pylori* antibody test results were negative upon admission. There was no history of *Helicobacter pylori* infection or eradication, and no history of long-term PPI use. Gastroscopy revealed that the gastric body mucosa was smooth, without rugae swelling, and there was white mucus present. The background showed RAC (regular arrangement of collecting venules). A lesion was found in the fundus of the stomach, classified as Paris classification type 0-IIa + Is, approximately 2.0 × 2.5 cm in size. The lesion appeared slightly whitish, with well-defined margins, a villous appearance, and central dot-like structures. Under Blue Light Imaging (BLI), the lesion appeared dark brown (Figures 1a–e). Magnifying endoscopy revealed that the background mucosa exhibited a normal gastric fundus mucosal structure, characterized by a regular honeycomb pattern and pit-like openings, and RAC pattern in the background mucosa surrounding. The lesion showed villous changes, with expanded and slightly distorted glandular structures that were irregularly arranged and exhibited significant structural atypia. The microvascular diameter was slightly increased and mildly distorted, demonstrating mild atypia (Figures 1f–l). After staining with indigo carmine and acetic acid, the atypical features of the lesion became more distinct, with well-defined borders and no evidence of intestinal metaplasia in the surrounding area. The lesion was considered to be a gastric-type tumor (Figures 1m,n). Endoscopic ultrasound indicated that the lesion was confined to the mucosal layer, with no enlargement of the perigastric lymph nodes (Figure 1o).

**Figure 2:**
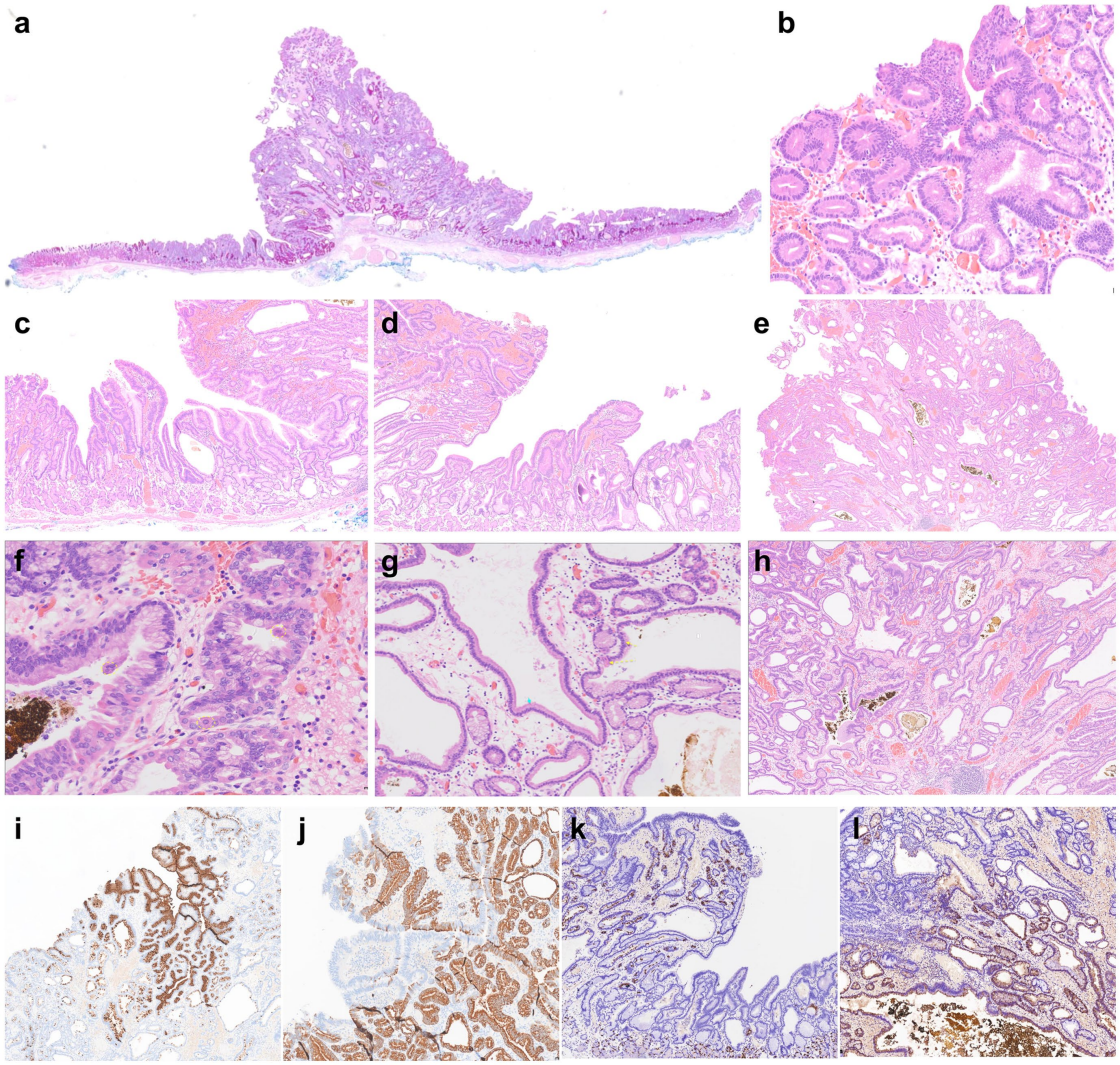
**(a)** Tumor was 0-IIa + Is under low magnification, located in the mucosal lamina propria without invasion of the mucosal muscle. **(b)** Under high magnification, the nuclei were diverse in shape and densely packed, with glands twisted. **(c,d)** The IIa region demonstrates mucous neck cell-type differentiation. **(e)** The Is region demonstrates a villous or papillary architecture with notable chief cell-type and parietal cell-type features. **(f)** High magnification reveals principal cells and parietal cells. **(g)** High magnification shows acinar epithelium differentiation cells and pyloric gland cells. **(h)** Dark brown pigmentation is observed in the lumen. **(i)** Immunohistochemical Muc-5 AC shows positivity. **(j)** Immunohistochemical Muc-6 shows positivity. **(k)** Immunohistochemical H+/K + ATPase shows partial positivity. **(l)** Immunohistochemical pepsinogen I marks gastric principal cells.

The patient underwent diagnostic endoscopic submucosal dissection (ESD). The pathological report showed the following findings: under low magnification, the tumor exhibited a 0-IIa + Is morphology, and the tumor tissue was confined to the mucosal propria without invading the muscularis mucosae (Figure 2a). Histological features: The nuclei exhibit various shapes and are densely packed, occupying nearly the entire layer, and the glandular structures are twisted (Figure 2b). For the IIa region (Figures 2c,d), the tumor exhibits a predominantly horizontal growth pattern. It is composed of densely packed, back-to-back or cribriform glands, primarily demonstrating mucous neck cell-type differentiation (resembling a pyloric gland adenoma). The neoplasm is confined to the superficial to middle mucosal layers, with an intact basement membrane. In the Is region (Figure 2e), the tumor exhibits a predominant vertical growth pattern forming a polypoid structure. The glands are elongated and branched, with a villous or papillary architecture. There is mixed differentiation, with notable chief cell-type and parietal cell-type features. Under high magnification, chief cells, parietal cells, foveolar epithelial cells, and pyloric gland cells are observed (Figures 2f–h). Immunohistochemistry: Muc-5 AC, strongly positive; foveolar epithelial cells, positive in some tumor areas; Muc-6, diffusely positive in the propria glands; and the H+/K + ATPase and pepsinogen I were positive (Figures 2i–l). The final diagnosis was a gastric-type tumor with multiple cell differentiation types, including foveolar, chief cell, and parietal cell types. The patient remained asymptomatic and stable during the 2-year follow-up period, with no evidence of recurrence or metachronous gastric cancer detected on gastroscopy performed at 6, 12, and 24 months after ESD.

## Case 2

**Figure 3:**
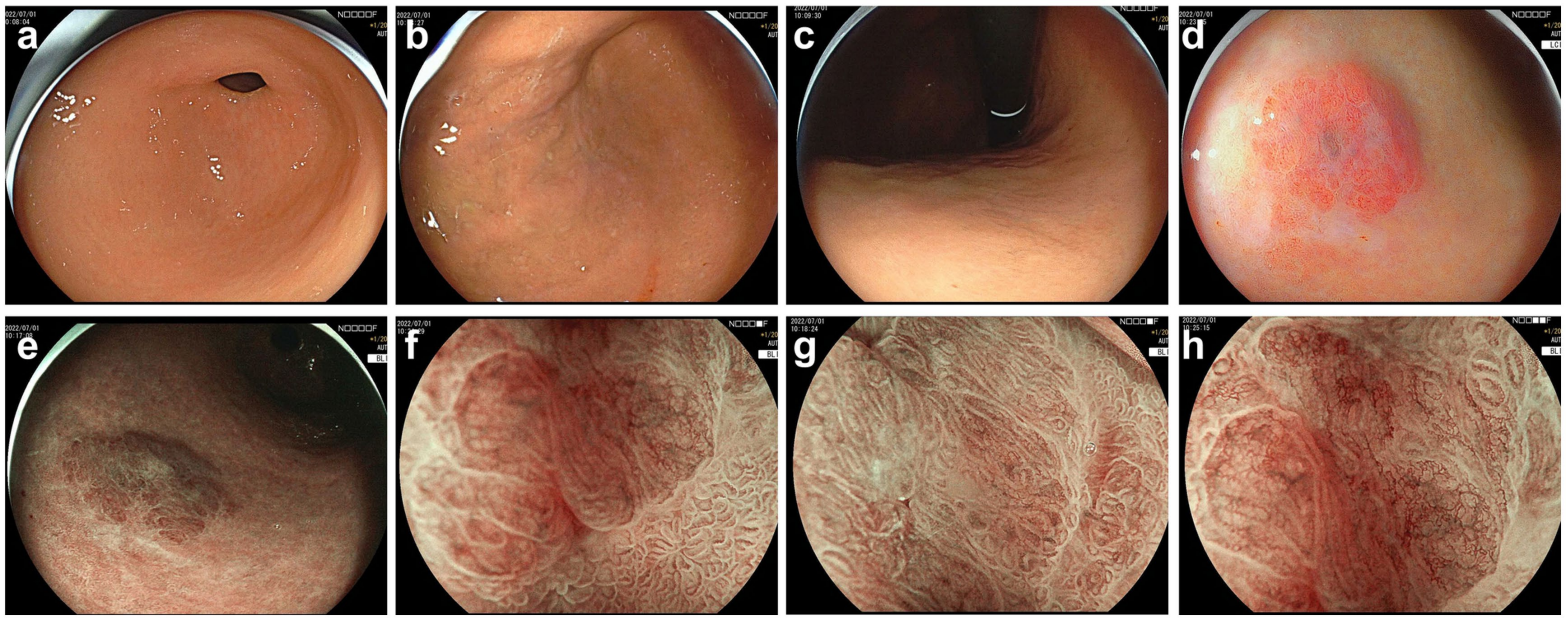
**(a)** Mucosa of the antrum of the stomach shows no atrophy. **(b)** White light revealed that the mucosa of the gastric body was smooth, without mucus adhering, and diffuse erythema. **(c)** The mucosa of the upper part of the gastric body was smooth. **(d)** Under LCI, the lesion appeared orange-yellow with an apricot background. **(e)** The lesion appears tea-brown under BLI. **(f-h)** Magnifying endoscopy reveals clear boundaries, dilated and tortuous glandular ducts with disordered arrangement, and fine-network-like microvessels of uniform diameter.

A 56-year-old woman presented with a chief complaint of upper abdominal pain for more than 2 months. She denies any family history of tumors. The C13 breath test was negative, she had never undergone *H. pylori* eradication treatment, and she had no history of long-term PPI use. Endoscopy revealed that the antrum mucosa exhibited a red-and-white pattern, with red predominating. The mucosa of the gastric body showed no diffuse redness. The mucus was clear, and the mucosa of the fundus was smooth (Figures 3a–c). Under white light endoscopy, a lesion of type 0-IIa was identified at the junction of the gastric antrum and body. The lesion measured approximately 0.6 × 0.6 cm in size and appeared slightly reddish. Under linked color imaging (LCI), it exhibited an orange-yellow hue and was lobulated, with visible patches of gray-brown discoloration and black dot-like structures. When viewed with BLI, the lesion appeared tea-brown. Magnifying endoscopy revealed distinct lesion borders, with dilated and distorted glandular ducts in a disorganized pattern. The microvasculature appeared as a fine network with uniform vessel diameters (Figures 3d–h).

**Figure 4:**
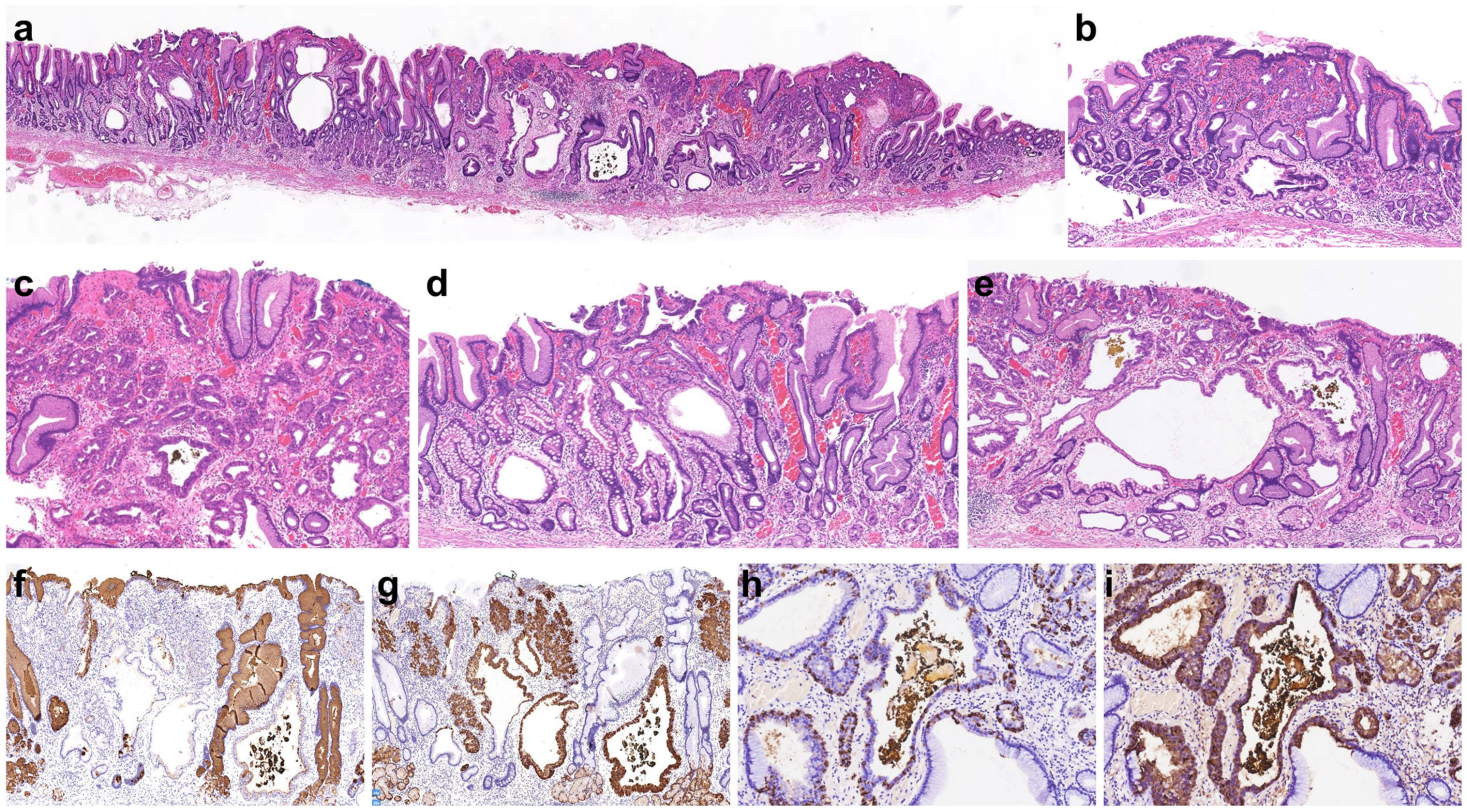
**(a)** Tumor and non-tumor areas were clearly demarcated under low magnification, located in the lamina propria of the mucosa without invading the mucosal muscle. **(b)** Under high magnification, the tumor cells have large and deeply stained nuclei, and the epithelial type tumor differentiation can be seen in the small sulcus. **(c)** High magnification microscopy reveals that the tumor cells were characterized by parietal cell and main cell differentiation. **(d)** High-power microscopy reveals that tumor cells beneath the superficial layer exhibit mucinous cervical cell differentiation and pyloric gland cell characteristics. **(e)** High-power microscopy indicates that tumor cells differentiate into parietal cells with dark brown pigment deposition in the lumen. **(f)** Immunohistochemical analysis shows Muc-5 AC positivity in the acinar epithelium. **(g)** Immunohistochemical Muc-6 shows positivity. **(h)** Immunohistochemical H+/K + ATPase shows partial positivity. **(i)** Immunohistochemical pepsinogen I marked gastric principal cells.

After ESD treatment, the pathological findings are as follows: The tumor tissue was confined to the upper and middle layers of the mucosa, with low-grade atypia. The small, depressed tumor epithelium was distributed in the surface area of the mucosa. The tumor tissue beneath the surface epithelium showed mucous neck cell differentiation with densely arranged glands, resembling features of pyloric gland adenoma. Some tumor tissues exhibited differentiation into chief cells and parietal cells but lacked the classic branched anastomosis structure characteristic of gastric fundic gland-type adenocarcinoma. In the mucosal upper-middle layer, the tumor glands exhibited cystic dilation, with the tumor epithelium showing differentiation into both chief cell-like and mucous neck cell-like types. Brown pigment substances could be seen deposited in the lumen, corresponding to the patchy gray-brown appearance visible through endoscopy (Figures 4a–e). Immunohistochemical staining showed that Muc-5 AC, Muc-6, H+/K + ATPase, and pepsinogen I were positive (Figures 4f–i). The final diagnosis was similar to that of case 1, which is also a gastric-type tumor with multiple cell differentiation types, including foveolar, chief, and parietal cell types. During the follow-up period, the patient underwent endoscopic evaluations at 6, 18, and 36 months after ESD, with no evidence of lesion recurrence or metastasis observed in any of these examinations.

## Discussion

With improvements in hygienic conditions and increased emphasis on health in China, more and more people are receiving eradication therapy for *H. pylori*. Consequently, the incidence of *H. pylori*-related gastric cancer is showing a gradual decline, while the relative proportion of HpNGC is increasing. HpNGC refers to gastric adenocarcinoma that occurs in the absence of current or past *H. pylori* infection (2, 8). Although several types of HpNGC have been reported (9), its etiology and diagnostic criteria remain unclear. Therefore, effective identification of different types of HpNGC is particularly important.

Negative *H. pylori* refers to the absence of current or past *H. pylori* infection, characterized by no history of *H. pylori* eradication, no evidence of *H. pylori* infection upon endoscopic and/or pathological evaluation, and negative results on urea breath tests (UBTs) and/or serum IgG tests. Endoscopic features of *H. pylori*-negative status include the absence of gastric mucosal atrophy and the presence of RAC, as well as possible findings of speckled redness and/or gastric fundic gland polyps. Pathological examination not only confirms the presence or absence of *H. pylori* infection but also assesses active inflammation, atrophy, and intestinal metaplasia (10, 11). Clinical diagnostic methods can detect *H. pylori* infection, but are not effective in distinguishing between current and past infections (12). Therefore, assessing *H. pylori* negativity, current *H. pylori* infection, and post-eradication gastritis, through endoscopic appearance and correlation with pathological results, is essential for comprehensive evaluation of *H. pylori* infection status.

The reported cases of HpNGC include six types: signet ring cell carcinoma (SRCC), gastric cancer of fundic gland type, small pit epithelial type gastric cancer, cardia adenocarcinoma, pyloric gland-type gastric cancer, and mixed gastric cancer (6, 7). SRCC predominantly occurs in young female patients and is often found as white, flat, or depressed lesions in the lower or middle part of the stomach. Compared with *H. pylori*-positive SRCC, *H. pylori*-negative SRCC originates from mucus neck cells and is less invasive (13, 14). Gastric cancer of the fundic gland type is commonly found in elderly patients and is characterized by differentiation toward acid-secreting glands. Magnification endoscopy with narrow-band imaging (ME-NBI) reveals abnormalities in microvessels and microstructures. Tumor cells typically differentiate toward chief cells and/or parietal cells and express pepsinogen I and/or H+/K ± ATPase (15). The small-pit epithelial-type gastric cancer is often observed endoscopically as lesions with a villous or papillary regular appearance, a whitish or decolorized appearance with lateral development, or a raspberry-like appearance. Pathologically, the surface may show atypical columnar cells with abundant cytoplasm, and the tumor cells resemble small pit epithelial differentiation with a papillary or villous structure. The expanded glands on the surface are made up of non-tumor cells pushing out tumor-like epithelium. Generally, the tumor is confined to the surface and is a low-grade differentiated cancer with low atypia. Immunohistochemical staining of the tumor tissue typically shows positivity for Muc-5 AC (16, 17). Cardia adenocarcinoma originates from the cardia glands and presents as red, depressed lesions. Narrow-band imaging (NBI) reveals structural abnormalities. Pyloric gland-type gastric cancer originates from the pyloric glands and shows positivity for CD10 and chromogranin A in immunohistochemistry. Mixed cell-type gastric cancer originates from various cell types, including small pit epithelial cells, fundic gland cells, pyloric gland cells, or intestinal-type cells (7).

The two cases of early gastric cancer in this article occurred in the background mucosa without *H. pylori* infection, and the mucosal microstructure and microvessels observed by magnification endoscopy combined with blue laser imaging (ME-BLI) were similar to those of ordinary gastric cancers, which could not be effectively differentiated endoscopically. Those two cases both were gastric-type tumors confined to the mucosa and showed immunohistochemical positivity for Muc-5 AC, Muc-6, H+/K + ATPase, and pepsinogen I, indicating multi-lineage differentiation (foveolar, chief, and parietal). More importantly, case 1 presented a 0-IIa + Is morphology with densely packed, highly atypical nuclei occupying the entire layer and twisted glands, while case 2 showed a small depressed lesion with tumor epithelium mainly in the upper/middle mucosa, featuring low-grade atypia, cystic dilation of glands, lumen pigment deposition, and a pattern resembling pyloric gland adenoma but lacking the classic branched anastomosis of fundic gland-type adenocarcinoma. However, case 1 aligns more with a prototypical gastric-type tumor showing overt architectural complexity and cytological atypia, whereas case 2 represents a lower-grade variant with distinct features that may correlate with indolent behavior. Clinically, we discuss how both cases, treated successfully with ESD and showing no recurrence at 2-year follow-up, support endoscopic resection as a viable strategy, while differences in grade and morphology underscore the importance of precise histopathological subclassification for prognosis and follow-up planning. The final diagnosis was based on histopathology and immunohistochemistry. Histologically, the superficial part of the lesion was positive for Muc-5 AC, with high cellular anisotropy and continuation to the submucosa, and combined with immunohistochemistry of Muc6 positivity, this tumor was thus determined to be a gastric-type tumor. Judging from the immunohistochemical Muc6, Muc-5 AC, pepsinogen I, and H+/K + -ATPase positivity, the carcinoma was characterized by multidirectional differentiation, with small concave epithelioid-type cells on the surface of the lesion, and predominantly cells at the base, mural cells, and mucus neck cells. Although a basic pathological feature of the gastric mucosa is the ability of multidirectional differentiation, the structure of early gastric carcinoma with a mixture of multiple cells is not rare and is completely different from the previously reported HpNGC (6, 7, 15).

Pyloric gland adenomas are typically composed of densely packed pyloric gland-like tubules, with cells showing mild to moderate atypia. Their characteristic immunophenotype is positive for MUC6, focally positive or negative for MUC5AC, and they are often associated with mutations in tumor suppressor genes. When the PGA (Pyloric Gland Adenoma) undergoes malignant transformation, cellular atypia increases significantly, and more complex architectures may appear, but it often retains some features of pyloric gland differentiation. The GA-FG in this case also forms tubular structures histologically, but its cell morphology more closely resembles normal fundic gland chief cells and parietal cells, with relatively bland cytologic atypia. A more crucial distinguishing point lies in the immunophenotype: GA-FG characteristically expresses chief cell markers (e.g., pepsinogen I) and parietal cell markers (e.g., H+/K + -ATPase) and consistently expresses MUC6, while MUC5AC is typically negative or only focally positive in rare cells.

For multidirectionally differentiated early gastric cancers, a distinction needs to be made between those that originate from pyloric gland adenomas. It is well known that Muc6 positivity is closely associated not only with the mucus neck cells of the basal glands but also with the pyloric glands, so effective differentiation is needed in clinical studies. Regarding the cellular origin of GA-FG, a hypothesis proposes that it may originate from the mucous neck cells of the gastric glands. The immunohistochemical findings observed in this case (i.e., tumor cells expressing MUC6 and focally expressing MUC5AC) are consistent with this hypothesis, as normal mucous neck cells also co-express these two mucins. Simultaneously, the morphology of the tumor cells resembles that of mucous neck cells in the proliferative zone in some areas. However, it must be noted with caution that the current evidence remains indirect and speculative. Similarities in immunophenotype and morphological analogy, while providing suggestive associations, do not constitute direct lineage evidence. The validation of this hypothesis requires future studies using more advanced techniques. On the one hand, the case in this article did not have a typical endoscopic presentation and histologic basis for the origin of pyloric adenoma (18, 19), and the epithelial region of the carcinoma was immunohistochemically Muc-5 AC-positive. In addition, the subepithelial component of pyloric adenomas may show low-grade atypical hyperplasia of the glands. The cases in this article were immunohistochemically Muc6-positive, and the Muc6-positive cells were of mucus neck cell origin rather than pyloric gland cells; therefore, the tumor cells in the two cases in this article were derived from gastric mucus neck cells.

Gastric mucous neck cells (MNCs) are distributed in the neck region of gastric fundus glands in small numbers, with a columnar shape, flat and deeply stained nuclei, and cytoplasm filled with glycogen granules. Studies have confirmed that MNCs exist in the neck of the fundic glands for approximately 7–14 days and have the ability for multidirectional differentiation and proliferation, and the abnormal proliferation and over-differentiation of MNCs can form gastric pre-cancerous lesions, which may ultimately lead to malignant tumors (20). Watanabe et al. reported, for the first time, the expression of Reg genes in gastric tissues (21). The Reg family is a group of proteins encoding a group of important proteins that are involved in the gastric mucosal response to injury and are currently thought to be responsible for the induction of MNCs. The Reg family encodes a group of important proteins involved in the response of the gastric mucosa to injury and is currently considered a unique growth factor that induces MNC differentiation and proliferation. Meanwhile, the abnormal or ectopic expression of Reg proteins and their receptors is closely related to the development of gastric cancer, which is expected to serve as a molecular target for the monitoring and treatment of gastric precancerous lesions and carcinomas (22, 23).

Thus, the Reg gene is involved in the damage response of the gastric mucosa, which contributes to the differentiation and proliferation of MNCs, leading to the development of mixed-cell-type early gastric cancer. This may be a pathological mechanism for the development of HpNGC. In this article, we report two rare cases of *H. pylori*-negative early gastric cancer, which is a new subtype of HpNGC. The following are the characteristic clinical and pathological manifestations: first, prominent shaped and/or minimally elevated reddish or whitish lesions, with distorted mucosal microstructures and irregular microvessels visible on magnified endoscopy; second, histology with pathological features of microconvex epithelial type and mucus neck cell type. The histology is characterized by small concave epithelial type, mucous neck cell type, master cell type, and mural cell type pathology.

## Data Availability

The raw data supporting the conclusions of this article will be made available by the authors, without undue reservation.
